# Laser-assisted hatching in lower grade cleavage stage embryos improves blastocyst formation: results from a retrospective study

**DOI:** 10.1186/s13048-021-00844-7

**Published:** 2021-07-15

**Authors:** Weihai Xu, Ling Zhang, Lin Zhang, Zhen Jin, Limei Wu, Shishi Li, Jing Shu

**Affiliations:** Department of Reproductive Endocrinology, Zhejiang Provincial People’s Hospital, Hangzhou Medical College, 310000 Hangzhou, P.R. China

**Keywords:** Blastocyst, Embryo, Zona pellucida (ZP), Laser-assisted hatching

## Abstract

**Background:**

Laser-assisted hatching (LAH) has been widely applied to facilitate blastocyst hatching in IVF-ET treatment, however, the effect of LAH on subsequent development and clinical outcomes of the lower grade cleavage stage embryos (LGCE) remains unknown. Our study aimed at evaluating the effect of LAH on blastocyst formation and the clinical pregnancy outcomes of LGCE embryos after transfer.

**Methods:**

A total of 608 cycles of IVF/ICSI treatment from November 2017 to September 2019 were included in our study as follows: 296 in the LAH group and 312 in the N-LAH group. The total blastocyst rate, usable blastocyst rate, good-grade blastocyst rate and clinical pregnancy rate were statistically compared between the two groups.

**Results:**

The total blastocyst rate (50.7% vs 40.2%, *P* < 0.001), usable blastocyst rate (31.0% vs 18.6%, *P* < 0.001) were significantly higher in the LAH group than those in the N-LAH group. After analysis of generalized estimating equations, LAH was positively correlated with the blastocyst rate (B = 0.201, OR 95% CI = 1.074–1.393, *P* = 0.002), usable blastocyst rate (B = 0.478, OR 95% CI = 1.331–1.955, *P* < 0.001). However, the clinical pregnancy rate after blastocyst transfer did not differ between LAH group and N-LAH group (49.4% vs 40.0%, *P* > 0.05, respectively).

**Conclusions:**

A higher proportion of total blastocysts and usable blastocysts can be obtained by LAH in LGCE, which may be beneficial to the outcome of the IVF/ICSI-ET cycle.

## Background

The best utilization of LGCE is still a challenge in conventional IVF and/or ICSI cycles with subsequent embryo transfer (IVF/ICSI-ET). During in vitro embryo culture, the most common types of LGCE include delayed cell division, unevenness of blastomeres and/or fragmentation. Due to insufficient mitochondrial content [1], increased probability of chromosomal abnormalities [2], and uneven distribution of ATP and mtDNA copy number between blastomeres [3], LGCE usually present with a considerably reduced probability of developing into an available blastocyst during conventional culture [4]. Although LGCE have a chance to establish a pregnancy after D3 transfer, it has been reported that the pregnancy rate is low [5]. Screening the embryos with high potential through extended culture of the LGCE, and transferring blastocysts can increase clinical pregnancy rate [6, 7]. However in conventional blastocyst culture it has been considered difficult to produce satisfactory outcome for LGCE. In order to improve the blastocyst development of LGCE, some studies have tried special techniques in practice, but the conclusions remain contradictory. Previous investigations have shown that the presence of fragments in LGCE are related to increased levels of reactive oxygen species (ROS) and apoptosis and thus impair embryonic development [8, 9]. Eftekhari-Yazdi et al. [10] removed the fragments from the embryo with severe fragmentation by a mechanical method, and reported that this technology can improve the embryo’s developmental potential, resulting in high-quality blastocysts. However, other authors proposed that this method can’t compensate for the loss of cytoplasmic components in blastomeres resulting from fragmentation, and hence is not able to improve the blastocyst formation rate. Even more, the extra loss of mitochondria during the defragmentation procedure might further reduce LGCE developmental potential [11, 12]. Therefore, there is so far no evidence-based method that can improve the development of LGCE and blastocyst formation. In treatment cycles where the outcome from transferring LGCE should be optimized, it is crucially important that a practicable and effective method is established which is able to improve the chances of obtaining a blastocyst.

LAH is a laser technology which uses infrared wavelength for drilling or thinning of the zona pellucida (ZP) to help embryos hatch [13]. It has been reported that LAH is unlikely to increase the clinical pregnancy rates when performed in transferred fresh embryos, but it has been proven that can produce good clinical results in the frozen-thawed embryo (FET) cycles [14, 15], which may be beneficial to the development of LGCE. Alikani et al. [16] have applied LAH and microsurgery to remove some or all fragments to restore the spatial relationship of cells within the embryo. Hershlag et al. [17] suggested that LAH provides an artificial passageway for the transportation of nutrients into embryos, which has been acknowledged as an important factor for their growth and development. Nevertheless, whether LAH can improve the utilization efficiency of LGCE and increase the probability of obtaining blastocysts remains to be proven. So we hypothesized that LAH is beneficial for embryos to eliminate toxins and intake nutrients, which is essential for the development of embryos and blastocysts formation.

## Methods

### Study subjects

This study was performed at the Reproductive Medicine Center of Zhejiang Provincial People's Hospital, Hangzhou Medical College from November 2017 to September 2019. To minimize confounding factors with respect to the observation endpoint, we established inclusion and exclusion criteria as follows: the inclusion criteria were (1) female, ≤ 45 years old, (2) blastocysts cultured from LGCE with normal fertilization, and (3) ≥ 4 blastomeres in each cleavage stage embryo; the exclusion criteria were: (1) chromosomal abnormalities in any partner of a couple, and (2) rescue ICSI cycles. From November 2017 to September 2019, 686 cycles met the inclusion criteria while 78 cycles were excluded for female chromosome abnormalities (n = 21), male chromosomal abnormalities (n = 16) and rescue ICSI (n = 41). Finally, a total of 608 cycles which contained 476 cycles with good quality embryos and LGCE, and 132 cycles with only LGCEs. 312 cycles without LAH were defined as N-LAH group, in which 57 cycles (≤ 38 years old) received a frozen blastocyst transfer, while 296 cycles with LAH on day 4 were defined as LAH group, in which 77 cycles (≤ 38 years old) received FET. Ovarian stimulation, oocyte retrieval, fertilization and culture conditions were similar between the two groups. This study was approved by the Reproductive Ethics Committee of Zhejiang Provincial People's Hospital, Hangzhou Medical College (Protocol #SZ2017011).

### In vitro fertilization, embryo grading and selection for transfer

All the controlled ovarian stimulation (COS) protocols were performed according to the standard procedure of our center which included antagonist protocol, long protocol, ministimulation scheme and others. When two follicles reached a diameter > 17 mm or one a follicular diameter > 18 mm, 5000 IU human chorionic gonadotropin (hCG) and/or 0.2 μg GnRH-a were injected to trigger oocyte maturation, and oocytes were retrieved 36 to 38 h thereafter. IVF or ICSI was performed 39 to 40 h after ovulation induction, the pronuclear (PN) check for fertilization was performed 16 to 18 h later. Embryos were cultured to D3 and were scored according to ASEBIR consensus [18] by two embryologists. In brief, Grade A: 7 ~ 8 cells with even symmetry, < 10% fragmentation, and no multinucleation; Grade B: 7 ~ 8 cells with even symmetry and 11 ~ 25% fragmentation, or ≥ 9 cells with even symmetry and < 26% fragmentation, and the LGCE were categorized except for Grade A and B. Embryos with multinucleation were not considered for this study. The LGCE embryos were remained in culture until D5. The formation of blastocysts and transformation of blastomeres were examined. The inner cell mass (ICM) and trophectoderm (TE) of blastocysts were graded using Gardner’s criteria [19]. In brief, Grade 1 (early blastocyst): blastocoele < 1/2 of total embryo volume; Grade 2 (intermediate blastocyst): blastocoele ≥ 1/2 of total embryo volume; Grade 3 (full blastocyst): blastocoele fully occupies the embryo; Grade 4 (expanded blastocyst): blastocoele is larger than early blastocyst and zona pellucida(ZP) demonstrates thinning; Grade 5 (hatching blastocyst): herniation of trophectoderm cells from the ZP; and Grade 6 (hatched blastocyst): blastocyst has escaped the ZP. For blastocysts at Grades 3 to 6, the inner cell mass (ICM) and trophectoderm (TE) were also graded. The ICM was graded as follows: A (many ICM cells packed together tightly); B (several ICM cells grouped loosely) and C (very few ICM cells). TE was graded as follows: A (many TE cells forming multiple epithelial layers); B (few TE cells consisting of a loose epithelium) and C (very few large TE cells). Blastocysts at Grade 3BB and above consisted of a visible and compacted ICM with a cohesive TE were considered as good grade blastocysts. Blastocysts at Grade 3 and above, one of ICM or TE was Grade C as sub-optimal blastocysts. Good grade blastocystsand sub-optimal blastocysts are defined as usable blastocysts.

All usable blastocysts were then vitrified for transfer in the subsequent FET cycles. One or two blastocysts were selected, thawed and transferred into individual patients following the endometrium preparation for FET. Clinical pregnancy was confirmed as the presence of a gestational sac and heart beat as detected by ultrasound 35 days after transfer. The absence of fetal heart beat or loss of pregnancy within 12 weeks after transfer was diagnosed as early abortion. A pregnancy developing beyond 12 weeks was classified as ongoing pregnancy.

### Embryo culture and LAH

All embryos were cultured in COOK media (COOK Medical, Brisbane, Australia). For LAH group, embryos were hatched on day 4 by using the Octax Laser Shot System (Vitrolife, Bruckberg, Germany). The laser parameters were as follows: near infrared diode wavelength 1480 nm, energy in focus 150 J and laser pulse length 2.6 ms. The zona pellucida was completely drilled by generating an opening of 10 μm in diameter. All LAH procedures were performed on D4. To avoid damaging the blastomere during operation, areas with a large perivitelline space between the blastomere and zona pellucida (ZP) or rich in fragments were selected for LAH.

### Statistical analysis

The main analytical parameters in the study included total blastocyst rate, good-grade blastocyst rate, usable blastocyst rate, sub-optimum blastocyst rate, and clinical pregnancy rate of FET. D5 blastocyst rate = Number of blastocysts formed in D5/ Number of LGCE cultured, D6 blastocyst rate = Number of blastocysts formed in D6/ Number of LGCE cultured and total blastocyst rate = Total number of blastocysts / Number of LGCE cultured. Data showing normal distribution were expressed as mean ± standard deviation (Mean ± SD), *t* and *χ*^*2*^*/F* test were used for comparing variables between the two groups. Generalized estimating equations was used to assess the effect of LAH on blastocyst formation and binary logistic regression was performed to analyze the contribution of LAH to clinical outcomes. A value of *P* < 0.05 was considered statistically significant. All statistical analysis was performed using SPSS (version 19.0).

## Result

### Characteristics of the study population

As shown in Table 1, there were no significant differences in patient’s age, infertility duration, basal follicle-stimulating hormone (D3 FSH), anti-müllerian hormone (AMH), history of pregnancy, infertility factors and COS protocols between the LAH group and N-LAH group. The fertilization rate was comparable in the two groups (*P* > 0.05) although the number of oocytes in the LAH group was higher than that in the N-LAH group (*P* < 0.05).

**Table 1:** Demographic parameters and fertilization rates in the two groups

Parameters	LAH group	N-LAH group	*P*
Cycles (n)	296	312	
No. of LGCE	948	808	
Female age (yrs)	32.2 ± 5.0	32.8 ± 5.4	0.121
Male age (yrs)	34.0 ± 5.6	34.4 ± 5.8	0.324
Infertility duration (yrs)	3.2 ± 2.6	3.1 ± 2.3	0.755
No. of treatment cycles	1.8 ± 1.4	1.8 ± 1.4	0.744
Basal FSH (IU/L)	6.2 ± 3.3	6.5 ± 5.4	0.425
AMH (IU/L)	3.4 ± 2.8	3.6 ± 3.0	0.388
BMI (kg/m^2^)	21.6 ± 3.0	22.0 ± 3.1	0.122
Primary /Secondary infertility	139/157	128/184	0.141
Infertility factors			0.126
Female factor	91	106	
Male factor	52	34	
Both factors	137	152	
Idiopathic factor	16	20	
COS procedure			0.107
Long protocol	45	48	
Antagonist protocol	174	167	
ministimulation scheme	60	62	
Others	17	35	
No. of oocyte	9.7 ± 6.0	8.1 ± 5.2	< 0.001^a^
Fertilization protocol (IVF/ICSI)	188/108	204/108	0.63
Fertilization rate (%)			
IVF	63.7(1174/1842)	67.3(1097/1630)	0.563
ICSI	72.0(511/710)	70.4(463/658)	0.512

*LAH* Laser assisted hatching, *N-LAH* Non laser assisted hatching, *LGCE* Low grade cleavage embryo, *COS* Controlled ovarian stimulation

^a^Statistical significance

### Comparison of blastocyst outcomes between the two groups

As shown in Table 2, the total blastocyst rate (50.7% vs 40.2%, *P* < 0.001), usable blastocyst rate (31.0% vs 18.6%, *P* < 0.001), good grade blastocyst rate (13.1% vs 9.2%, *P* = 0.009) and sub-optimum blastocyst rate (17.9% vs 9.4%, *P* < 0.001) in the LAH group were significantly higher than those in the N-LAH. Further comparison of the blastocyst formation revealed that there was a higher usable blastocyst rate (14.8% vs 9.5%, *P* < 0.001) and sub-optimum blastocyst rate (7.2% vs 3.9%, *P* = 0.003) on D5 in the LAH group as compared to those in the N-LAH group. There were significant differences in total blastocyst rate (16.2 and 9.1%, *P* < 0.001), sub-optimum blastocyst rate (10.7 and 5.5%, *P* < 0.001) between the LAH and N-LAH Groups on Day 6. We employed the generalized estimating equations to assess the effect of LAH on blastocyst formation after adjusting for female age, BMI, oocyte number and fertilization protocol. As showed in Table 3. LAH is positively correlated with blastocyst rate (B = 0.201, OR 95% CI = 1.074–1.393, *P* = 0.002), usable blastocyst rate (B = 0.478, OR 95% CI = 1.331–1.955, *P* < 0.001), but has no significantly difference in good grade blastocyst rate.

**Table 2:** Outcomes of blastocyst culture in LAH and N-LAH groups

		LAH	N-LAH	*P*
Cycles		296	312	
No. of LGCE		948	808	
Blastocyst rate (%)	D5	27.8(264/948)	19.7(159/808)	> 0.05
	D6	22.9(217/948)	20.5(166/808)	> 0.05
	Total	50.7(481/948)	40.2(325/808)	< 0.001
Usable blastocyst rate (%)	D5	14.8(140/948)	9.5(77/808)	< 0.001
	D6	16.2(154/948)	9.1(74/808)	< 0.001
	Total	31.0(294/948)	18.6(151/808)	< 0.001
Good grade blastocyst rate (%)	D5	7.7(73/948)	5.7(46/808)	> 0.05
	D6	5.4(51/948)	3.5(28/808)	> 0.05
	Total	13.1(124/948)	9.2(74/808)	0.009
Sub-optimum blastocyst rate (%)	D5	7.2(68/948)	3.9(32/808)	0.003
	D6	10.7(101/948)	5.5(44/808)	< 0.001
	Total	17.9(170/948)	9.4(76/808)	< 0.001

*LAH* Laser assisted hatching, *N-LAH* Non laser assisted hatching

**Table 3:** The related factors influence the blastocyst formation

	B	S.E	Sig	OR	95% CI	
					Lower	Upper
Blastocyst formation						
Female age	-0.013	0.007	0.073	0.987	0.973	1.001
BMI	-0.030	0.012	0.009	0.97	0.949	0.993
No. of oocyte	0.013	0.005	0.004	1.013	1.004	1.022
LAH performed	0.201	0.066	0.002	1.223	1.074	1.393
Fertilization protocol	-0.116	0.068	0.089	0.891	0.780	1.018
Usable blastocyst formation						
Female age	-0.015	0.010	0.149	0.985	0.966	1.005
BMI	-0.045	0.016	0.006	0.956	0.927	0.987
No. of oocyte	0.014	0.007	0.038	1.014	1.001	1.027
LAH performed	0.478	0.098	< .0001	1.613	1.331	1.955
Fertilization protocol	-0.265	0.105	0.012	0.767	0.624	0.943
Good blastocyst formation						
Female age	-0.012	0.017	0.464	0.988	0.956	1.021
BMI	-0.054	0.026	0.037	0.947	0.900	0.997
No. of oocyte	0.042	0.009	< .0001	1.043	1.025	1.060
LAH performed	0.251	0.155	0.107	1.285	0.948	1.742
Fertilization protocol	-0.145	0.167	0.383	0.865	0.624	1.199

The outcomes of blastocyst culture were also compared in a sub-population of 132 cycles without good grade embryos on D3, 48 cycles of LAH and 84 cycles without LAH were also compared. There was higher usable blastocyst rate (22.2% vs 9.2%, *P* = 0.002) and sub-optimum blastocyst rate (15.4% vs 5.4%, *P* = 0.004) in the LAH group compared to those the N-LAH group. Moreover, there was significant difference in the usable blastocyst rate (12.8% vs 4.9%, *P* = 0.013) and sub-optimum blastocyst rate (11.1% vs 3.2%, *P* = 0.006) on D6 between LAH and N-LAH groups as shown in Table 4.

**Table 4:** Outcomes of blastocyst culture in LAH and N-LAH groups in cycles without good grade embryo

		LAH	N-LAH	*P*
Cycles		48	84	
No. of LGCE		117	185	
Blastocyst rate (%)	D5	16.2(19/117)	11.8(22/185)	> 0.05
	D6	17.1(20/117)	16.2(30/185)	> 0.05
	Total	33.3(39/117)	28.1(52/185)	> 0.05
Usable blastocyst rate (%)	D5	9.4(11/117)	4.3(8/185)	> 0.05
	D6	12.8(15/117)	4.9(9/185)	0.013
	Total	22.2(26/117)	9.2(17/185)	0.002
Good grade blastocyst rate (%)	D5	5.1(6/117)	2.2(4/185)	> 0.05
	D6	1.7(2/117)	1.6(3/185)	> 0.05
	Total	6.8(8/117)	3.8(7/185)	> 0.05
Sub-optimum blastocyst rate (%)	D5	4.3(5/117)	2.2(4/185)	> 0.05
	D6	11.1(13/117)	3.2(6/185)	0.006
	Total	15.4(18/117)	5.4(10/185)	0.004

*LAH* Laser assisted hatching, *N-LAH* Non laser assisted hatching

**Fig. 1:**
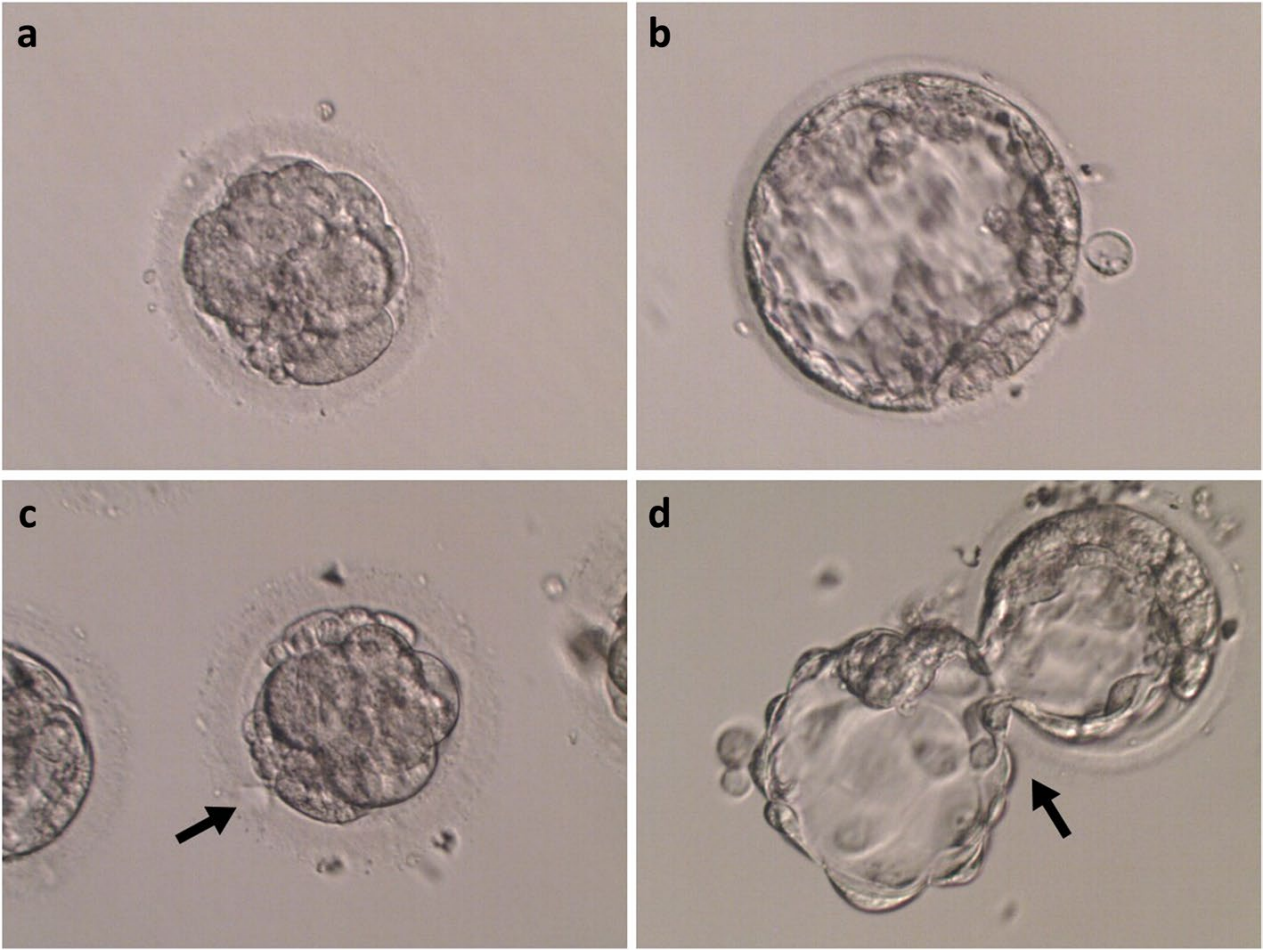
The comparison of morphological characteristics of blastocyst between LAH and N-LAH groups. **a** LGCE containing high proportions of fragments without LAH on D4; **b** D6 blastocyst from the N-LAH group with obscure structure of the inner cell mass (ICM) and trophoblast cells; **c** LGCE containing a high proportion of fragment with LAH on D4; **d** D6 blastocyst from the LAH group which was not fully expanded when hatching, and the inner cell mass and trophoblast cell structure were distinct and integral. The arrow shows the position of LAH

During the blastocyst culture of LGCE, the dark coloration and/or fragmentation of blastomeres were the most common morphological phenotypes, which often deteriorated with progressing culture time, as shown in Fig. 1. There was significantly lower proportion of the dark coloration and fragmentation on D6 blastocysts in LAH group compared to that in the N-LAH group (34.2% vs 49.6%, *P* < 0.001), although there was no significant difference in the proportion of dark coloration and fragmentation on D5 between groups (3.2% vs 3.7%, *P* > 0.05), as shown in Table 5.

**Table 5:** The morphological alteration in blastocyst culture

	LAH	N-LAH	*P*
No. of LGCE	948	808	
The rate of dark coloration and fragmentation on D5 (%)	3.2(30/948)	3.7(30/808)	> 0.05
The rate of dark coloration and fragmentation on D6 (%)	34.2(324/948)	49.6(401/808)	< 0.001

*LAH* Laser assisted hatching, *N-LAH* Non laser assisted hatching, *LGCE* Low grade cleavage embryo

### Correlation between embryo transfer outcome and LAH

A total of 134 FET cycles were performed with blastocysts originating from LGCE, resulting in 61 clinical pregnancies (45.5%). Analysis with binary logistic regression model revealed that transfer of the good grade blastocyst transfer as the only significant factor influencing the pregnancy outcome of the transfer cycles (95% OR = 1.528–5.739, *P* = 0.001), while other factors, such as the LAH treatment (95% OR = 0.296–1.472, *P* = 0.310) and blastocyst age (95% OR = 0.143–1.129, *P* = 0.084) did not alter the clinical pregnancy rate, as shown in Table 6.

**Table 6:** The related factors influence the outcome of blastocyst transfer

	B	S.E	Sig	OR	95% CI	
					Lower	Upper
Female age	-0.096	0.054	0.079	0.909	0.817	1.011
BMI	-0.075	0.073	0.308	0.928	0.804	1.071
History of pregnancy	-0.172	0.420	0.682	0.842	0.370	1.916
Insemination protocol	-0.262	0.435	0.547	0.770	0.328	1.804
Blastocyst age	-0.913	0.528	0.084	0.401	0.143	1.129
No. of blastocyst	0.281	0.397	0.478	1.325	0.609	2.884
No. of good grade blastocyst	1.086	0.338	0.001	2.961	1.528	5.739
LAH performed	-0.415	0.409	0.310	0.660	0.296	1.472

*LAH* Laser assisted hatching, *BMI* Body mass index, *B* Regression coefficient, *S.E*. Standard error, *Wals* Wald statistics, *Sig*. Statistical significance, *OR* Odds ratio

The clinical outcomes following embryo transfer were summarized in Table 7. There were no differences in implantation rate, clinical pregnancy rate, miscarriage rate and delivery rate between the LAH Group and N-LAH Group (*P* > 0.05). Moreover, the birth rate in the LAH group was similar to that in the N-LAH group (*P* > 0.05). No congenital malformations were observed in both groups. These results suggested that assisted hatching with laser under the designated parameters in this study does not affect the clinical pregnancy and live birth rates.

**Table 7:** Clinical outcome in LAH and N-LAH groups

	LAH	N-LAH	*P*
Cycles (n)	77	57	
Female age (yrs)	31.0 ± 4.1	31.4 ± 3.9	0.61
Blastocyst transferred	1.5 ± 0.5	1.5 ± 0.5	0.829
Good blastocyst transferred	0.9 ± 0.7	0.8 ± 0.7	0.613
Clinical pregnancy rate (%)	49.4(38/77)	40.4(23/57)	0.381
Implantation rate (%)	37.1(43/116)	32.2(28/87)	0.47
Miscarriage rate (%)	15.8(6/38)	8.7(2/23)	0.698
Number of delivery	22	19	
Ongoing pregnancy	10	2	
Number of newborns	27	23	
Birth weight (g)	3308 ± 265	3280 ± 572	0.87
LBW	2	4	
Congenital malformations	0	0	

*LAH* Laser assisted hatching, *N-LAH* Non laser assisted hatching, *LBW* low birth weight

There was no significant difference in clinical pregnancy, implantation and ongoing pregnancy rates with transfer of good grade blastocyst between LAH and N-LAH groups (*P* > 0.05). The clinical outcomes with transfer of sub-optimum blastocysts were also comparable between the two groups (*P* > 0.05) as shown in Table 8.

**Table 8:** Clinical outcome of different quality blastocysts

	Transfer Good grade blastocyst								Transfer sub-optimum blastocyst					
	Cycles (n)	Age (yrs)	No. of blastocyst	PR (%)	IR (%)	MR (%)	OPR (%)	Cycles (n)	Age (yrs)	No. of blastocyst	PR (%)	IR (%)	MR (%)	OPR (%)
LAH group	40	30.5 ± 4.3	1.4 ± 0.5	67.5(27/40)	54.7 (29/53)	18.5 (5/27)	55.0	25	31.9 ± 4.2	1.5 ± 0.5	24.0 (6/25)	16.2 (6/37)	1/6	20.0
N-LAH group	29	31.6 ± 3.6	1.3 ± 0.5	51.7(15/29)	45.9 (17/37)	6.7 (1/15)	48.3	19	30.3 ± 4.3	1.6 ± 0.5	15.8 (3/19)	12.9 (4/31)	1/3	10.5
*P*		0.242	0.472	0.218	0.413	0.293	0.581		0.238	0.327	0.710	0.701		0.566

*LAH* Laser assisted hatching, *N-LAH* Non laser assisted hatching, *PR* pregnancy rate, *IR* implantation rate, *MR* miscarriage rate, *OPR* ongoing pregnancy rate

## Discussion

Our study aimed at evaluating the effect of LAH on blastocyst formation and the clinical pregnancy outcomes of LGCE embryos after transfer. Our data showed that LAH treatment on D4 resulted in significantly higher proportions of total blastocysts and usable blastocysts, and transfer of these blastocysts in LAH group produced similar clinical outcomes, including implantation, clinical pregnancy and miscarriage when compared to those of N-LAH group (*P* > 0.05). Moreover, there were no significant in differences in ongoing pregnancy and live birth rates between the LAH and N-LAH groups (*P* > 0.05), suggesting the safety of LAH on embryos at morula stage on D4. To our knowledge, this is the first clinical study on the effect of LAH on the outcome of blastocysts developed from LGCEs.

Our results were in accordance with previous studies showing that culture of LGCE to blastocysts may improve the clinical outcomes rate per transfer [6, 20] and the cumulative delivery rate [21]. Shaw-Jackson et al. [6] reported that vitrification of blastocysts derived from 914 LGCEs produced high pregnancy rates after warming. Fifty blastocyst warming cycles resulted in a 76% survival rate, 44% clinical pregnancy rate and 39% implantation rate which were significantly higher than those in the cycles with D3 embryo transfer (*P* < 0.05). Kaartinen et al. summarized the cumulative live birth rate of 604 FET cycles [21]. The transfer of blastocysts derived from culture of LGCEs raised cumulative delivery rate from 43 to 47% and 53 repeated new cycles were avoided. This study shows that the cumulative delivery rate can be increased, and repeated IVF-ICSI treatments can be avoided by using blastocysts developing from poor-quality cleavage stage embryos, which otherwise would have been discarded.

Previous studies also revealed that LAH of embryos prior to transfer enhanced the clinical pregnancy rates per transfer cycle. Li et al. [22] summarized the clinical pregnancy outcomes of 6459 transfer cycles of 36 randomized clinical studies and concluded that the odds ratio was 1.16 in the LAH group which suggested that LAH improved the clinical pregnancy outcomes. González-Ortega et al. [23] reported that LAH of embryos in 303 IVF cycles with poor prognosis resulted in increased implantation rate when compared to that of N-LAH group (17.5% vs. 8.3%, *P* < 0.05). Jelinkova et al. [24] demonstrated that there was significant increase in implantation rate in the group with removal of zona pellucida of slow-growth embryos at morula or blastocyst stages compared to that of no removal of pellucida group (25.7% vs. 12.1%, *P* < 0.05). Our study extended previous studies that LAH of LGCEs on day 4 resulted in significant increase in usable blastocyst rate when compared to those of N-LAH (*P* < 0.05). Transfer of these blastocysts in FET cycles resulted in similar clinical pregnancy and live birth rates. There were no differences in implantation rate, clinical pregnancy rate, miscarriage rate and delivery rate between the LAH Group and N-LAH Group (*P* > 0.05). Moreover, the birth rate in the LAH group was similar to that in the N-LAH group (*P* > 0.05). No congenital malformations were observed in either groups.

The other studies, however, concluded that LAH on multiple-cell stage embryos failed to improve blastocyst formation and clinical pregnancy rate [25, 26]. Wong et al. reported that 114 donated blastomere stage embryos were divided into three groups: complete LAH, partial LAH and N-LAH. When LAH was performed on D2 or D3, there was no significant difference in blastocyst rate among the complete LAH, partial LAH and N-LAH groups (*P* > 0.05). Yano et al. also reported that a small group D3 embryos (*n* = 56) were assigned randomly to a zona opening group, a zona thinning group, or a control group. The blastocyst rate did not differ significantly between the zona opening and control groups (*P* > 0.05). In these studies, LAH was performed either on D2 or D3 which didn’t show improvement of blastocyst formation and clinical pregnancy rate. In the current study, however, we performed the LAH on D4 which may be benefit for the blastocyst development in the current study. There is more space and clear boundary between the compacted cells and the zona pellucida on D4, which may avoid potential damage of the embryos and warrants the safety with LAH. It has been well documented that the transition of maternal to zygotic control of transcription occurred at the 6–8 stage and completed at the morula stage of human embryos [27]. Thus, performing LAH on D4 may avoid interference of the genomic activation and development of these embryos and is relatively safe to avoid potential damage.

The beneficial effect of LAH on the development of LGCE may also be attributed to the following factors: beneficial for compaction of embryos and blastocyst formation, facilitating the diffusion of ROS out from embryo and related reduction of intra-blastocyst oxidative stress; alleviating the spatial oppression caused by fragmentation during compaction and formation of the morula [16]; reducing the probability of contact between the trophoblast and undesirable substances by promoting early hatching from the zona pellucida, and subsequently protecting the vitality and potential of the blastocyst; building an artificial pathway for nutrient transfer to the embryo, which is essential for the development of embryos [17]. During blastocyst development, apoptosis is an important pathophysiological factor compromising the vitality of blastomeres and the process of blastocyst formation from LGCE. Evidence from animal experiments has shown that prolonged embryo culture may increase the apoptosis level, reduce the ICM/TE ratio and disrupt the gene expression of blastocysts [28], while heavy fragmentation is accompanied with higher apoptotic cell ratios and embryo stagnation [29]. In the present study, LAH treatment has been observed to ameliorate the structural or morphological alteration including cell darkening and blastomeres fragmentation, and resulted in an increase of blastocyst formation, indicating that LAH is beneficial to the development of blastocysts.

### Limitations

Our data showed that LAH contributed to a better rate of blastocyst to LGCE. Indeed, there are some limitations in our study. The number of oocytes differed between the groups, although it did not change the conclusion of the study. The study divided group into LAH and N-LAH based on different time periods. Further prospective randomized study, and multiple-center, randomized clinical trial with larger samples are required to confirm the clinical benefit of the LAH of LGCE on D4.

## Conclusion

In the present study, LAH was conducted on LGCE on D4 resulted in significant increase in usable of blastocyst compared to those of N-LAH group (*P* < 0.05). The clinical outcome of blastocyst transfer in FET cycles in the LAH group was similar to that of N-LAH group (*P* > 0.05). Our data revealed that there were more transfer opportunities and a lower cancellation rate with LAH on D4 LGCEs, suggesting an efficient means for improving blastocyst development and avoiding repeat cycles in patients with cycles with poor quality embryos only.

## Data Availability

The raw data supporting the conclusions of this manuscript will be made available by the authors, without undue reservation, to any qualified researcher.

## Abbreviations

LAH: Laser-assisted hatching
LGCE: Lower grade cleavage stage embryos
ZP: Zona pellucida
COS: Controlled ovarian stimulation
ICM: Inner cell mass
TE: Trophectoderm
FET: Frozen-thawed embryo transfer
AMH: Anti-müllerian hormone
LBW: Low birth weight
ROS: Reactive oxygen species

## References

[1] Lin DP, Huang CC, Wu HM, Cheng TC, Chen CI, Lee MS (2004). Comparison of mitochondrial DNA contents in human embryos with good or poor morphology at the 8-cell stage. Fertil Steril.

[2] Hardarson T, Hanson C, Sjögren A, Lundin K (2001). Human embryos with unevenly sized blastomeres have lower pregnancy and implantation rates: indications for aneuploidy and multinucleation. Hum Reprod.

[3] Kameyama Y, Ohnishi H, Shimoi G, Hashizume R, Ito M, Smith LC (2010). Asymmetrical allocation of mitochondrial DNA to blastomeres during the first two cleavages in mouse embryos. Reprod Fertil Dev.

[4] O’Leary T, Duggal G, Lierman S, Van den Abbeel E, Heindryckx B, De Sutter P. The influence of patient and cohort parameters on the incidence and developmental potential of embryos with poor quality traits for use in human embryonic stem cell derivation. Hum Reprod. 2012;27(6):1581–9. 10.1093/humrep/des040 22442247

[5] Kirillova A, Lysenkov S, Farmakovskaya M, Kiseleva Y, Martazanova B, Mishieva N, Abubakirov A, Sukhikh G (2020). Should we transfer poor quality embryos?. Fertil Res Pract.

[6] Shaw-Jackson C, Bertrand E, Becker B, Colin J, Beaudoin-Chabot C, Rozenberg S, Autin C (2013). Vitrification of blastocysts derived from fair to poor quality cleavage stage embryos can produce high pregnancy rates after warming. J Assist Reprod Genet.

[7] Sallem A, Santulli P, Barraud-Lange V, Foll NL, Ferreux L, Maignien C, Bourdon M, Chapron C, de Ziegler D, Wolf JP, Pocate-CherietK. Extended culture of poor-quality supernumerary embryos improves ART outcomes. J Assist Reprod Genet, 2018; 35(2): 311–9. 10.1007/s10815-017-1063-7 PMC5845035 29047006

[8] Chi HJ, Koo JJ, Choi SY, Jeong HJ, Roh SI (2011). Fragmentation of embryos is associated with both necrosis and apoptosis. Fertil Steril.

[9] Yang HW, Hwang KJ, Kwon HC, Kim HS, Choi KW, Oh KS (1998). Detection of reactive oxygen species (ROS) and apoptosis in human fragmented embryos. Hum Reprod.

[10] Eftekhari-Yazdi P, Valojerdi MR, Ashtiani SK, Eslaminejad MB, Karimian L (2006). Effect of fragment removal on blastocyst formation and quality of human embryos. Reprod Biomed Online.

[11] Keltz M, Fritz R, Gonzales E, Ozensoy S, Skorupski J, Stein D (2010). Defragmentation of low grade day 3 embryos resulted in sustained reduction in fragmentation, but did not improve compaction or blastulation rates. Fertil Steril.

[12] Halvaei I, Khalili MA, Esfandiari N, Safari S, Talebi AR, Miglietta S, Nottola SA (2016). Ultrastructure of cytoplasmic fragments in human cleavage stage embryos. J Assist Reprod Genet.

[13] Minh Tam Le , Thi Tam An Nguyen, Thi Thai Thanh Nguyen, Van Trung Nguyen, Dinh Duong Le, Vu Quoc Huy Nguyen, et al. Thinning and drilling laser-assisted hatching in thawed embryo transfer: A randomized controlled trial. Clin Exp Reprod Med, 2018; 45(3):129–34. 10.5653/cerm.2018.45.3.129 PMC6125152 30202743

[14] Martins WP, Rocha IA, Ferriani RA, Nastri CO (2011). Assisted hatching of human embryos: a systematic review and meta-analysis of randomized controlled trials. Hum Reprod Update.

[15] Valojerdi MR, Eftekhari-Yazdi P, Karimian L, Ashtiani SK (2008). Effect of laser zona pellucida opening on clinical outcome of assisted reproduction technology in patients with advanced female age, recurrent implantation failure, or frozen-thawed embryos. Fertil Steril.

[16] Alikani M, Cohen J, Tomkin G, Garrisi GJ, Mack C, Scott RT (1999). Human embryo fragmentation in vitro and its implications for pregnancy and implantation. Fertil Steril.

[17] Hershlag A, Feng HL (2005). Effect of prefreeze assisted hatching on postthaw survival of mouse embryos. Fertil Steril.

[18] Alpha Scientists in Reproductive, M. and E.S.I.G.O. Embryology, The Istanbul consensus workshop on embryo assessment: proceedings of an expert meeting. Hum Reprod, 2011; 26(6): 1270–83. 10.1093/humrep/der037 21502182

[19] Gardner DK, Schoolcraft WB, Jansen R, Mortimer D (1999). In vitro culture of human blastocysts. Toward Reproductive Certainty: Fertility and Genetics Beyond 1999.

[20] Balaban B, Urman B, Alatas C, Mercan R, Aksoy S, Isiklar A (2001). Blastocyst-stage transfer of poor-quality cleavage-stage embryos results in higher implantation rates. Fertil Steril.

[21] Kaartinen N, Das P, Kananen K, Huhtala H, Tinkanen H (2015). Can repeated IVF-ICSI-cycles be avoided by using blastocysts developing from poor-quality cleavage stage embryos?. Reprod Biomed Online.

[22] Li D, Yang DL, An J, Jiao J, Zhou YM, Wu QJ, Wang XX (2016). Effect of assisted hatching on pregnancy outcomes: a systematic review and meta-analysis of randomized controlled trials. Sci Rep.

[23] González-Ortega C, Cancino-Villarreall P, Anaya-Torres FJ, Pérez-Peña E, Gutiérrez-Gutiérrez AM (2015). Impact of laser-assisted hatching (quarter technique) in poor prognosis patients. Ginecol Obstet Mex.

[24] Jelinkova L, Pavelkova J, Strehler E, Paulus W, Zivny J, Sterzik K (2003). Improved implantation rate after chemical removal of the zona pellucid. Fertil Steril.

[25] Wong BC, Boyd CA, Lanzendorf SE (2003). Randomized controlled study of human zona pellucida dissection using the zona infrared laser optical system: evaluation of blastomere damage, embryo development, and subsequent hatching. Fertil Steril.

[26] Yano K, Kubo T, Ôhashi I, Yano C. Assisted hatching using a 1.48-microm diode laser: Evaluation of zona opening and zona thinning techniques in human embryos. Reprod Med Biol, 2006; 5(3): 221–6. 10.1111/j.1447-0578.2006.00145.x PMC5906858 29699251

[27] Braude P, Bolton V, Moore S (1988). Human gene expression first occurs between the four- and eight-cell stages of preimplantation development. Nature.

[28] Lin T, Lee JE, Oqani RK, Kim SY, Cho ES, Jeong YD (2017). Delayed blastocyst formation or an extra day culture increases apoptosis in pig blastocysts. Anim Reprod Sci.

[29] Antunes G, Chaveiro A, Santos P, Marques A, Jin HS, Moreira da Silva F. Influence of apoptosis in bovine embryo's development. Reprod Domest Anim. 2010; 45: 26–32. 10.1111/j.1439-0531.2008.01131.x 19055557

